# Rotational alignment using a rotatable sphincterotome for difficult biliary cannulation of an intradiverticular papilla

**DOI:** 10.1055/a-2976-9129

**Published:** 2026-10-29

**Authors:** Hidenobu Hara, Kei Onodera, Ami Yoshinaga, Kotaro Amano, Ryota Nakamura, Kouhei Yoshino, Shinya Sakita

**Affiliations:** 1 Department of Gastroenterology 53327 Yokohama City Minato Red Cross HospitalYokohamaKanagawaJapan

Although selective biliary cannulation represents an essential step in endoscopic retrograde cholangiopancreatography (ERCP), failure may occur in up to 18% of cases. However, when performed by experienced practitioners, failure can be reduced to ≤5%. 1​ For patients with an intradiverticular papilla, owing to restricted endoscope maneuverability, difficulty may be experienced in identifying the papillary orifice and alignment of the cannulation device with the bile duct axis. 2​ However, in such anatomically challenging situations, a rotatable sphincterotome can facilitate biliary cannulation ( Fig. 1 ). 3​ 4​ ​5​ We present a case illustrating how rotational control of a sphincterotome can overcome axis mis-alignment during biliary cannulation of an intradiverticular papilla ( Video 1 ).

**Fig. 1:**
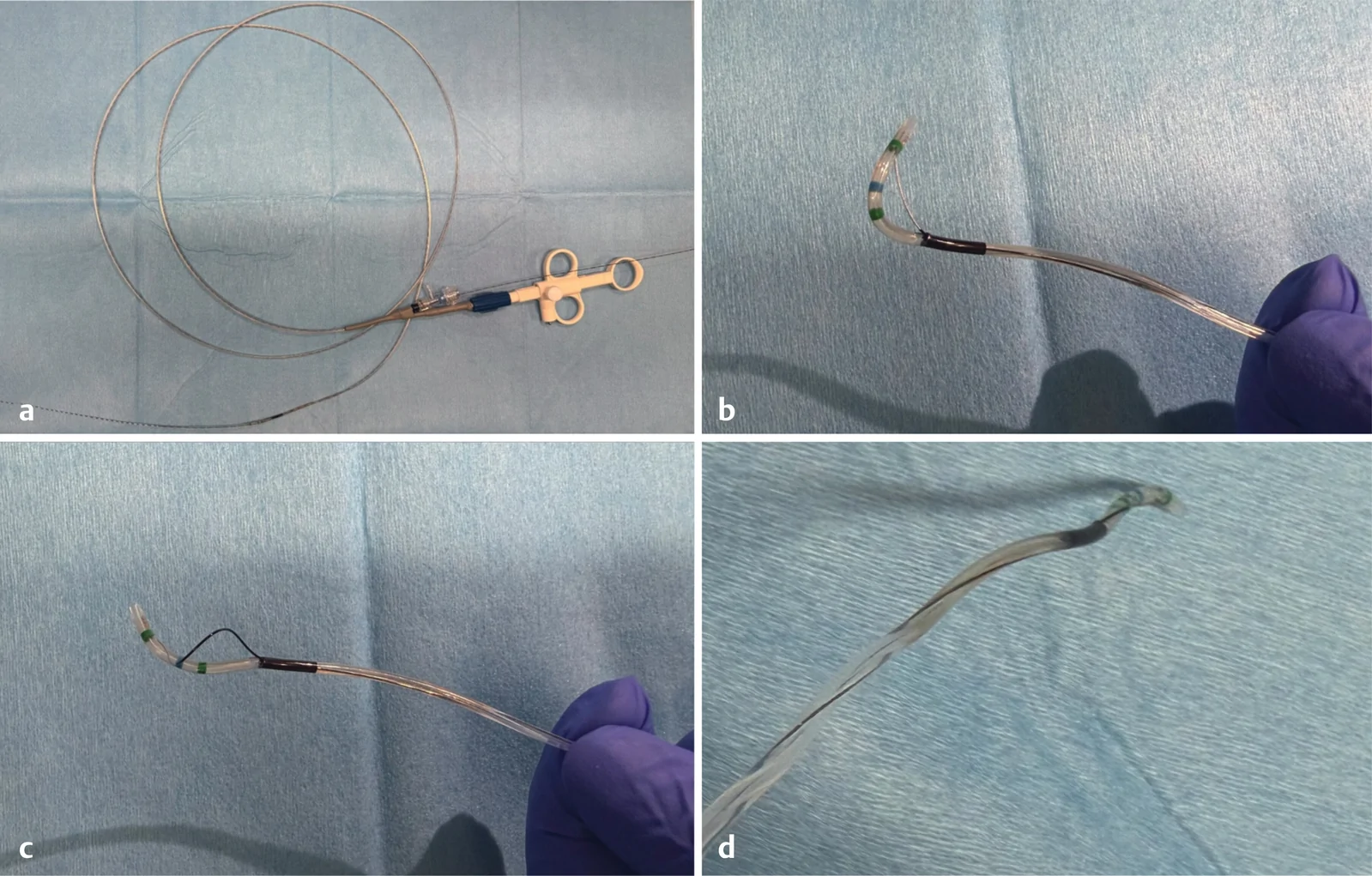
The rotatable sphincterotome used in this procedure. ( **a** ) The rotatable sphincterotome (ENGETSU; Kaneka Medix Corp., Osaka, Japan) is compatible with a 0.025-inch guidewire and has a working length of 2000 mm. ( **b** ) Pulling the handle adjusts blade angulation, with a maximum pull movement of 30 mm. ( **c** ) Pushing the handle adjusts blade angulation, with a maximum push movement of 15 mm. ( **d** ) The handle ring enables 360° clockwise or counterclockwise rotation, providing multidirectional control of the sphincterotome tip.

**Video 1** Rotational control of a sphincterotome for difficult biliary cannulation of an intradiverticular papilla. The video illustrates the identification of an intradiverticular papilla, unsuccessful cannulation with a standard catheter, device manipulation using blade angulation and rotation, and successful biliary cannulation following counterclockwise rotation of the sphincterotome.


An 83-year-old man was admitted with acute cholangitis caused by common bile duct stones. During ERCP, the major papilla could not be identified from the conventional endoscopic position ( Fig. 2a, b ), and cannulation of the suspected papillary region with a catheter and a guidewire proved unsuccessful ( Fig. 2c ). A duodenoscope was therefore carefully advanced into the diverticulum, facilitating the direct visualization of the papilla ( Fig. 3a ). However, a standard catheter could not be aligned with the bile duct axis ( Fig. 3b ).

**Fig. 2:**
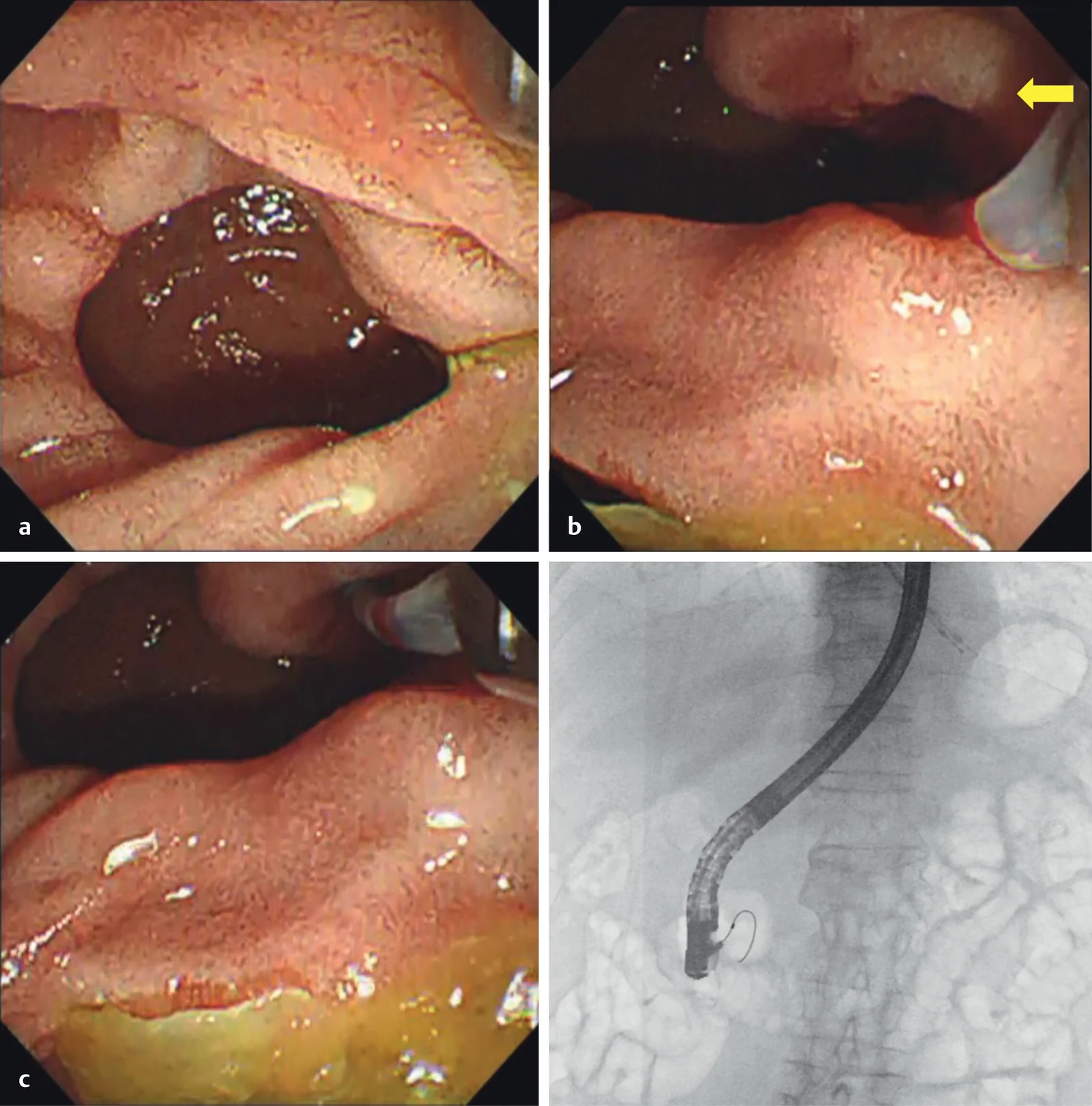
Endoscopic and fluoroscopic findings prior to advancement of the duodenoscope into the diverticulum. ( **a** ) The major papilla could not be identified from the conventional endoscopic position. ( **b** ) Upward angulation of the duodenoscope and probing of the suspected papillary region (arrow) with a catheter failed to achieve adequate visualization of the papilla. ( **c** ) Selective biliary cannulation using a standard catheter and a guidewire was unsuccessful.

**Fig. 3:**
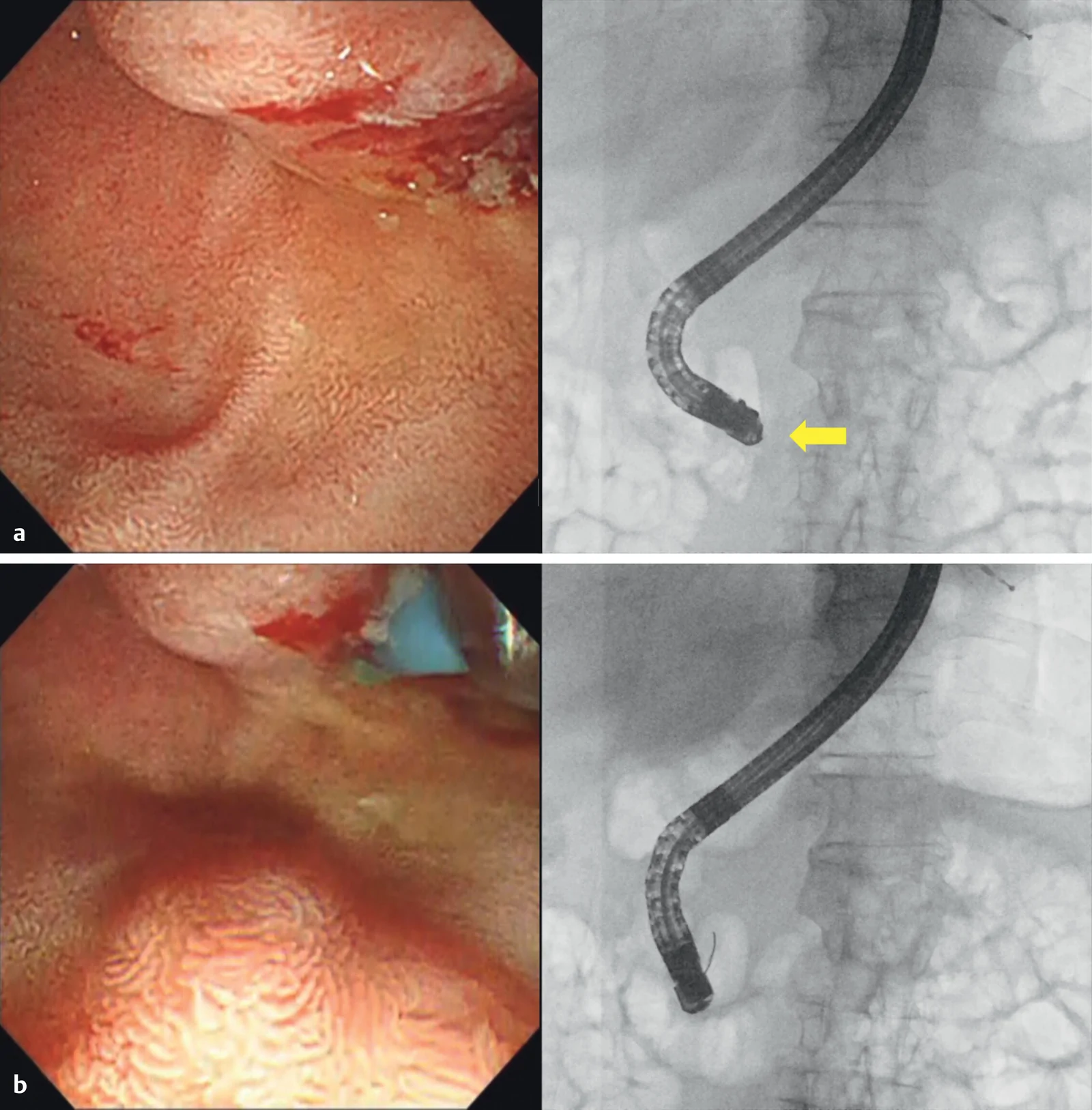
Endoscopic and fluoroscopic findings following advancement of the duodenoscope into the diverticulum. ( **a** ) Careful advancement of the duodenoscope into the diverticulum enabled the direct visualization of the intradiverticular papilla. The arrow indicates the duodenoscope tip within the diverticulum. ( **b** ) Cannulation with a standard catheter remained unsuccessful owing to difficulty in aligning the catheter with the bile duct axis.

Although the catheter was replaced with a rotatable sphincterotome and blade angulation directed the tip toward the bile duct, this failed to achieve sufficient coaxial alignment, owing to the restricted endoscope position within the diverticulum ( Fig. 4a ). Counterclockwise rotation subsequently aligned the sphincterotome with the bile duct, enabling successful cannulation and guidewire passage ( Fig. 4b ). Biliary drainage was completed with stent placement ( Fig. 4c ), without procedure-related adverse events.

**Fig. 4:**
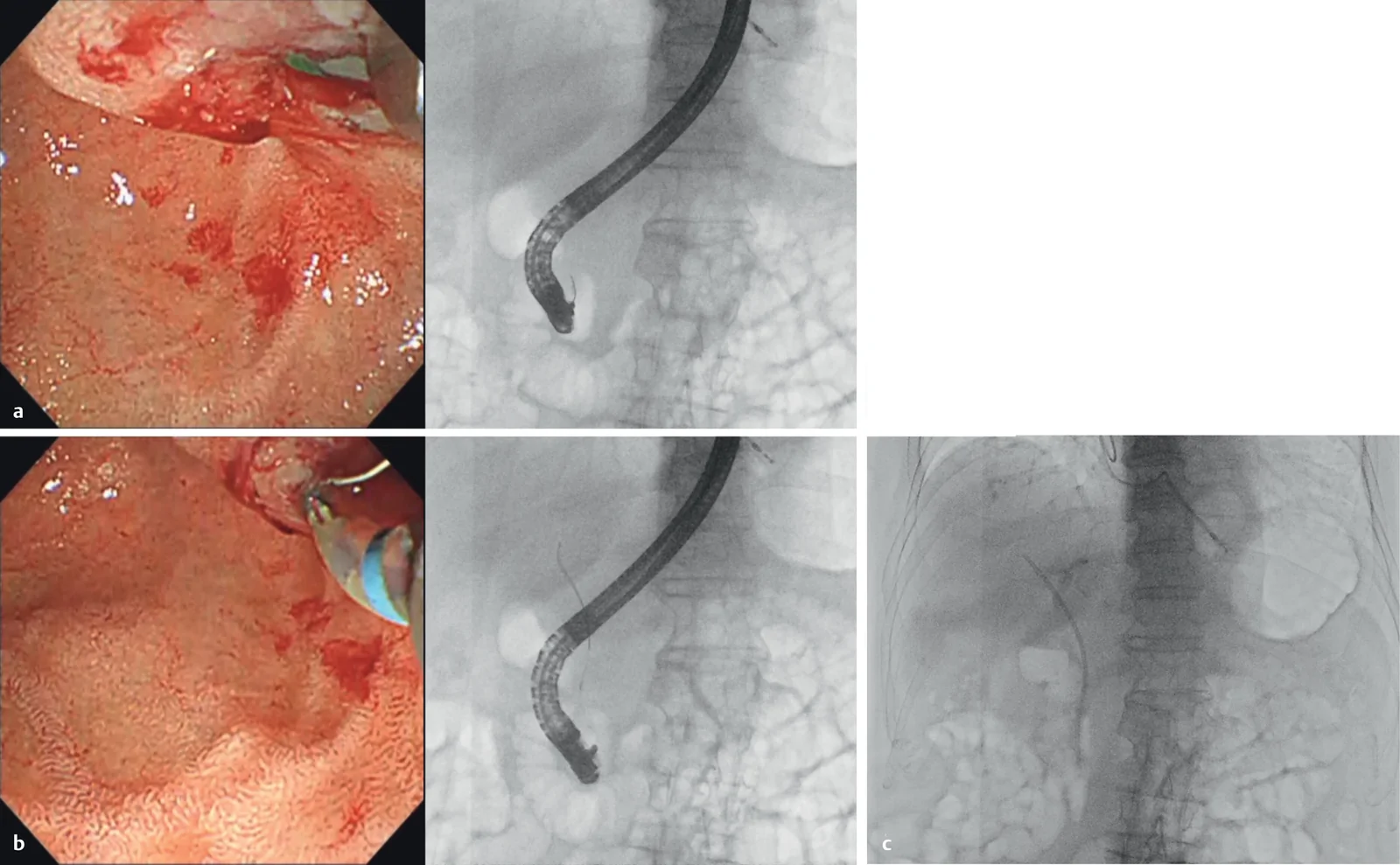
Endoscopic and fluoroscopic findings during biliary cannulation using the rotatable sphincterotome. (a) Although blade angulation directed the tip of the sphincterotome toward the papillary orifice, this was ineffective in facilitating sufficient coaxial alignment for deep cannulation and guidewire advancement. (b) Counterclockwise rotation of the sphincterotome facilitated coaxial alignment with the bile duct, enabling successful deep biliary cannulation and guidewire passage. (c) Biliary drainage was completed with the insertion of a 7-Fr, 10-cm plastic stent.

In intradiverticular papillae, a restricted working space may hinder device alignment. When blade angulation alone is insufficient, additional rotation of a sphincterotome may facilitate coaxial alignment and biliary cannulation, providing an additional degree of directional control when endoscope repositioning is limited.

Endoscopy_UCTN_Code_TTT_1AR_2AC

## Declaration of GenAI Use

The authors confirm that no generative artificial intelligence was used in the preparation of this manuscript.
